# Aggressive Angiomyxoma of the Vulva: A Bizarre Perineal Lesion

**DOI:** 10.1155/2015/292304

**Published:** 2015-04-22

**Authors:** Adamantia Zizi-Sermpetzoglou, Despoina Myoteri, Kalliroi Koulia, Vassilios Kontostolis, Hippokratis Moschouris, Dionysios Dellaportas

**Affiliations:** ^1^Pathology Department, “Tzaneion” General Hospital, 185 36 Piraeus, Greece; ^2^2nd Department of Surgery, “Tzaneion” General Hospital, 185 36 Piraeus, Greece; ^3^Radiology & Interventional Radiology Department, “Tzaneion” General Hospital, 185 36 Piraeus, Greece; ^4^2nd Department of Surgery, University Hospital “Aretaeion”, 115 28 Athens, Greece

## Abstract

*Introduction*. Aggressive angiomyxoma is a rare, slowly growing, and benign tumour of mesenchymal origin, which affects women of reproductive age and is associated with a high risk of local recurrence. *Case Presentation*. A case of a 47-year-old white female is presented herein, with a large polypoid, gelatinous mass on the right labia majora, measuring 26 × 21 × 6 cm. Histopathologically, the lesion was composed of spindle and stellate-shaped cells embedded in a myxoid matrix. Another specific feature was the presence of variable-sized thin-walled capillaries and thick-walled vascular channels. The patient underwent wide local excision of the tumour with clear margins and developed local recurrence 18 months later. *Discussion*. Aggressive angiomyxoma of the vulva needs to be distinguished from benign myxoid tumors with a low risk of local recurrence as well as from malignant myxoid neoplasms. Usually wide local excision with tumour-free margins and occasionally hormonal manipulation is the treatment of choice.

## 1. Introduction

Aggressive angiomyxoma (AA) is a rare, slowly growing, and benign tumour of mesenchymal origin. AA was described by Steeper and Rosai in 1983 as a tumour with a predilection for the perineum of reproductive-age women [1]. Very few cases have also been described in men, usually involving the scrotum. Female to male ratio is 6.6/1 and the term “aggressive” underlines the tumour's locally infiltrative nature, but mostly the high rate of local recurrence [2]. There is lack of agreement among pathologists regarding the tumour pathogenesis; however, a fibroblastic/myofibroblastic origin seems most likely [3]. A 47-year-old white female with a polypoid vulva lesion is presented herein, which turned out as AA, and a short description of the clinicopathological characteristics of this rare entity is also attempted.

## 2. Case Presentation

**Figure 1:**
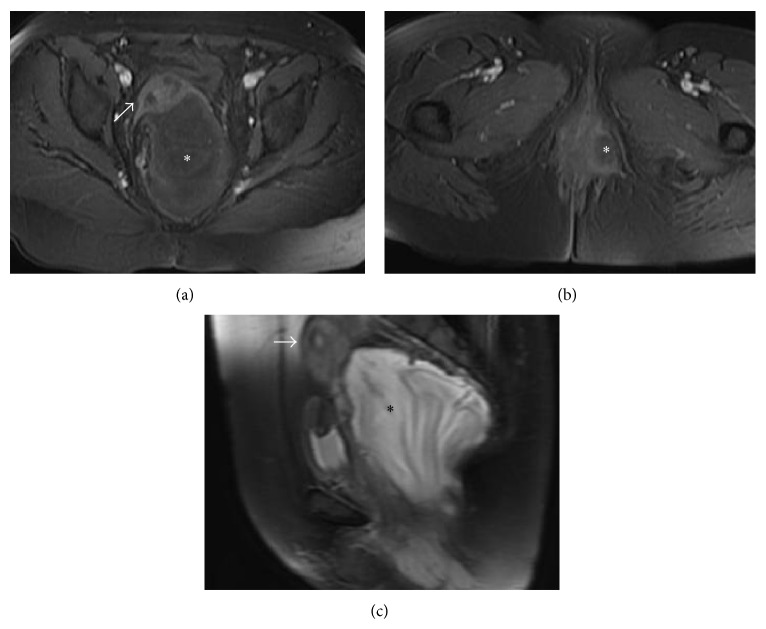
MR images of the lesion. (a, b) Axial, T1-weighted images with gadolinium enhancement and fat saturation. (c) Sagittal, T2-weighted image. The lesion (asterisk) has low signal on T1W images and high signal on T2W image. There is minimal contrast enhancement. The lesion causes anterior displacement of the uterus (arrows (a, c)). On (b), the downward extension of the lesion into the ischiorectal fossa is evident.

A 47-year-old female was admitted to our hospital with an enormous polypoid mass on her right labia majora initially noticed three years ago. The mass has gradually increased in size. On clinical examination a well-circumscribed polypoidal mass measuring approximately 25 × 20 × 6 cm was revealed. The lesion was soft and gelatinous in consistency, covered by normal appearing skin. The tumour was painless with no associated lymphadenopathy. On gynaecological examination the cervix and vagina were unremarkable. Her laboratory investigations were within normal limits and ultrasonography of the abdomen had nothing to add. Moreover, ultrasonography of the perineum showed a large mass with peripheral vascularity, heterogenous hyperechoic areas, measuring approximately 26 × 21 × 6 cm, and magnetic resonance (MRI) of the pelvis confirmed these features (Figures 1(a), 1(b), and 1(c)). The initial differential diagnosis included the fibroepithelial polyp, the vulval fibroma, or the giant acrochordon, and the patient underwent a wide local excision of the tumour. The procedure was complicated with profuse bleeding, but the postoperative course was uneventful.

**Figure 2:**
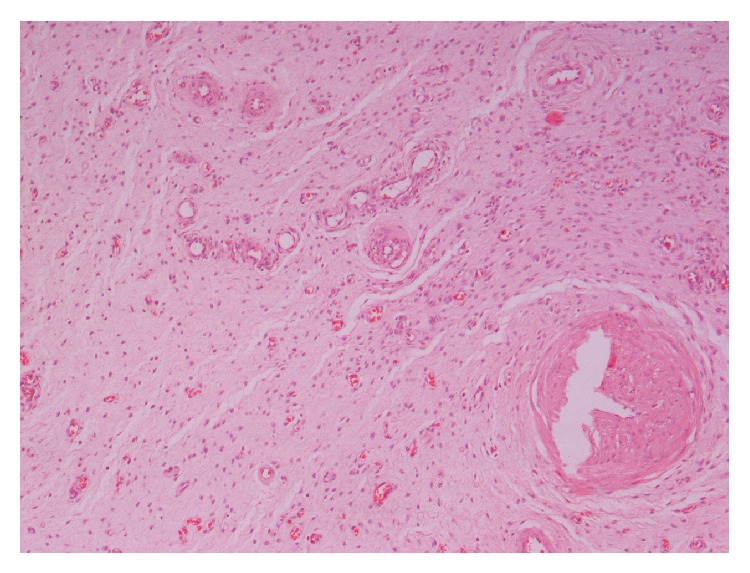
Spindle and stellate-shaped cells embedded in a myxoid matrix. Presence of variable-sized thin-walled capillaries and thick-walled vascular channels (H-E ×100).

**Figure 3:**
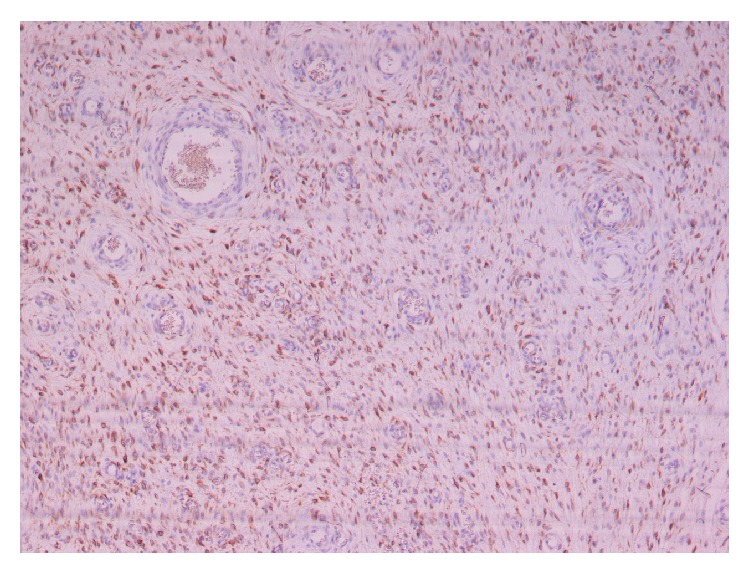
Tumor cells positive for desmin (desmin ×200).

**Figure 4:**
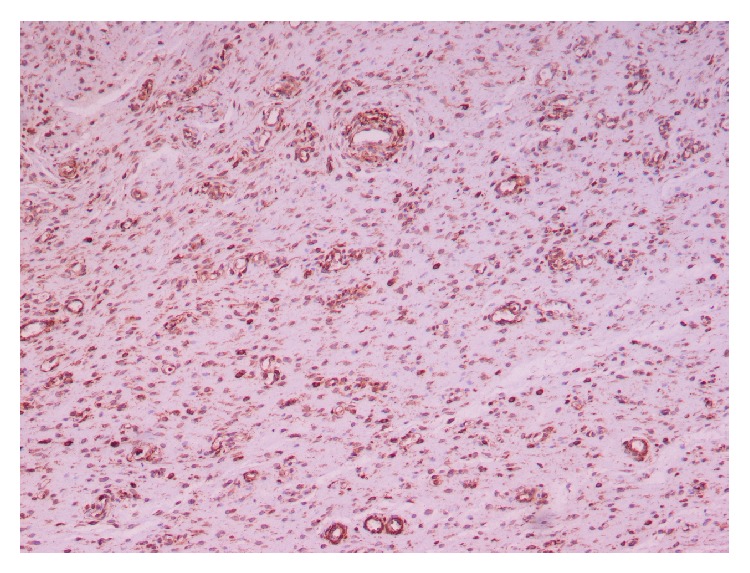
Tumor cells positive for vimentin (vimentin ×200).

Histopathological examination revealed an encapsulated neoplasm composed of stellate and spindled cells, embedded in a loose matrix with wavy collagen. The lesion contained thin-walled capillaries and thick-walled vascular channels. Neoplastic cells had relatively scant eosinophilic cytoplasm and their nuclei were ovoid, containing finely dispersed chromatin with one or two small eosinophilic nucleoli (Figure 2). There was no significant nuclear pleomorphism nor mitoses observed. Immunohistochemically, the tumour cells were positive for vimentin, actin, desmin, and progesterone but negative for S100 protein, CEA, CKAE1/AE3, factor VIII (Figures 3 and 4). The background stroma stained positive with alcian blue (pH 2.5). Combining all the above the diagnosis of AA is set.

The patient developed local recurrent disease 18 months later, which was revealed on her follow-up visits.

## 3. Discussion

In general, angiomyxomas are classified either as superficial (also known as cutaneous myxoma) or AA. Superficial angiomyxoma may occur in the setting of Carney complex [4]. This lesion is observed predominantly in male middle-aged adults and can arise anywhere in the superficial tissues, but mostly it involves the trunk, lower extremities, and head and neck. Clinically, most lesions appear as slowly growing polypoid cutaneous lesions and are easily confused with a cyst, skin tag, or neurofibroma.

AAs affect almost exclusively the genital, perineal, and pelvic regions of reproductive-age females and especially the vulva. Rarely AAs are reported in men, and when they do the scrotum is usually involved. Although growing slowly, these lesions behave “aggressively” by infiltrating the perivaginal and perirectal tissues and are characterized by very high rates of local recurrence [5].

The imaging characteristics of this tumour are interesting. On computed tomography (CT) scan, a circumscribed mass with attenuation less than or equal to that of adjacent skeletal muscle is the norm, with swirled enhancing tissue internally [6]. On MRI scan, this tumour shows high signal intensity on T2-weighted images and typically gives the same as in CT swirling appearance. Both features are likely to be related to high water content and the loose myxoid matrix of AA [7].

The main theory for AA pathogenesis is that a primitive multipotent mesenchymal cell of the lower female genital tract that has the capability of differentiating by various ways gives rise to the lesion. The latter is mainly supported by immunohistochemical expression of desmin and, in some cases, a smooth muscle actin along with desmin by tumour cells [8].

Interesting molecular studies have linked a consistent clonal aberration of the chromosome 12, in the region 12q13–15, associated with rearrangement of the HMGIC gene (high-mobility group protein isoform I-C) with AA. The study claims that AA is molecularly part of the benign group of mesenchymal tumours showing multiple aberration region involvement [9, 10]. HMGIC expression in AA is of value in the histological differential diagnosis.

Macroscopically, AA is a soft, well-defined, sometimes polypoid mass, ranging in size from a few centimetres to 20 cm or even more, as in our case. The main feature is a shiny, homogenous, gelatinous appearance on gross sections [11]. Microscopically, the tumour is composed of widely scattered spindled to stellate-shaped cells with ill-defined cytoplasm and small round to oval hyperchromatic nuclei with small centrally located nucleoli, embedded in a myxoid stroma. The latter is rich in collagen and often contains hemorrhagic foci. A defining special feature is the presence of variably sized vessels that range from small thin-walled capillaries to large vessels with secondary changes including perivascular hyalinization and medial hypertrophy. Immunohistochemically, AA cells show diffuse staining for vimentin and desmin. Immunostains for muscle-specific actin and smooth muscle actin are also positive in most cases as in all mesenchymal lesions, whereas S-100 protein and cytokeratins are negative [12]. Another feature of AAs is the consistent expression of both estrogen and progesterone receptors for both genders, suggesting connection with hormones on growth of these lesions [13].

The greatest clinical problem of AA is its high propensity for local recurrence. That is why differential diagnosis includes other benign myxoid neoplasms, such as angiomyofibroblastoma, intramuscular and juxta-articular myxoma, cutaneous myxoma (superficial angiomyxoma), myxoid neurofibroma, cellular angiofibroma, myxoid leiomyoma, and fibroepithelial stromal polyps [14]. On the other hand, AA may become enormously large and has an infiltrating tendency for the surrounding tissues, and as a result, low-grade myxoid sarcomas as well as myxofibrosarcoma, myxoid variant of liposarcoma, leiomyosarcoma, malignant fibrous histiocytoma, and botryoid rhabdomyosarcoma should be included in the differential diagnosis.

Therapeutic strategy relies on surgical approach with wide local excision with clear margins. The operative decision for adjacent organs, such as the rectum and bladder to which the tumour may be attached, is to spare them, as the morbidity of extensive surgery may not be justified. Local recurrences are treated with reoperation when possible and cannot be predicted neither from size nor from tumour cellularity. Recurrences are reported from months to several years after excision [15]. In our case the recurrent mass was revealed 18 months later on her follow-up and the plan is to proceed with reoperation.

Radiation therapy and chemotherapy have no practical implications due to low mitotic activity. Hormonal manipulation with tamoxifen, raloxifene, and gonadotropin-releasing hormone analogues has been tried and results in downsizing of the tumour in the neoadjuvant setting [16]. Long-term follow-up includes MRI for detecting recurrences as the most effective imaging modality [17].
